# Telomere Shortening in Interstitial Lung Disease: Challenges and Promises

**DOI:** 10.1111/crj.70103

**Published:** 2025-07-08

**Authors:** Haonan Jin, Jiamin Song, Ronglin Gao, Bingxian Sha, Shengyuan Wang, Peiming Luo, Li Yu, Xianghuai Xu, Xuan Wang

**Affiliations:** ^1^ Department of Pulmonary and Critical Care Medicine, Tongji Hospital, School of Medicine Tongji University Shanghai China; ^2^ Department of Rheumatology and Immunology, Tongji Hospital, School of Medicine Tongji University Shanghai China

**Keywords:** connective tissue disease, interstitial lung disease, pulmonary fibrosis, telomerase, telomere

## Abstract

Interstitial lung disease (ILD) is a group of diseases involving diffuse pulmonary parenchymal lesions and alveolar inflammation and interstitial fibrosis. Telomeres are repetitive DNA sequences at the end of chromosomes to maintain structural integrity and telomerase can prevent telomere shortening. Telomerase abnormalities such as related gene mutations lead to decrease in telomerase activity and telomere shortening. It has been proven that telomere shortening and telomerase abnormalities are related to the occurrence and development of ILD. Telomere shortening occurs in different types of ILD patients and is associated with prognosis. Gene therapy targeting telomerase exhibits therapeutic potential. The paper elaborates on the progress of telomere shortening in the diagnosis, differential diagnosis, treatment, and prognosis of ILD in recent years, in order to demonstrate its potential and promises and to be helpful for clinical diagnosis and treatment.

## Graphical abstract

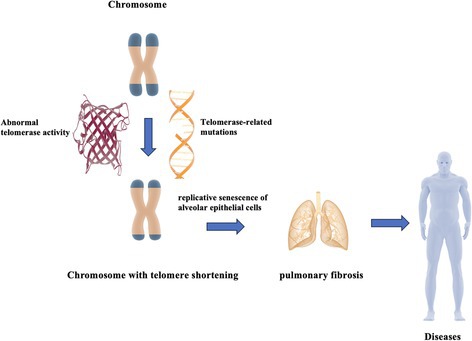

## 1 Introduction

Interstitial lung disease (ILD) is a heterogeneous group of diseases characterized by diffuse parenchymal lung lesions, alveolar inflammation, and interstitial fibrosis. The main types of ILD include idiopathic pulmonary fibrosis (IPF), connective tissue disease (CTD) related ILD, and granulomatous ILD. Among them, IPF, CTD‐ILD, and sarcoidosis account for more than half of all ILD cases [1]. ILD is incurable, and patients suffer from respiratory distress, limited mobility, reduced quality of life, and eventually death due to respiratory failure. In IPF, for example, the median survival period is 2–5 years with a 5‐year survival rate of 20%–40% [2, 3]. Except for a few rare ILDs with unique pathogenesis, the pathogenesis of most ILDs is complex and involves a variety of genetic, immunological, and environmental factors, such as telomere dysfunction, mitochondrial dysfunction, DNA damage, epigenetic alterations, inflammatory response, and protein homeostasis imbalance [4]. The pathology of ILD is a combination of lung inflammation and pulmonary fibrosis [5]. Studies have confirmed that the pathogenesis of pulmonary fibrosis is associated with telomerase mutations and telomere shortening, which are thought to be possibly related to telomere‐induced replicative senescence of alveolar epithelial cells. It has been demonstrated that telomeres are significantly shortened in all ILD patients [6]. The paper elaborates on the progress of telomere shortening in the diagnosis, differential diagnosis, treatment, and prognosis of ILD in recent years in order to demonstrate its potential and promises and to be helpful for clinical diagnosis and treatment.

Telomeres are complex structures at the ends of eukaryotic chromosomes composed of many simple repetitive sequences and associated proteins that serve to maintain the structural integrity of chromosomes and resolve their terminal replication challenges. Telomere shortening directly impairs epithelial proliferation and replication capacity, thereby contributing closely associated with cellular senescence and related diseases. Telomerase is an enzyme synthesized mainly by germ cells that stimulates DNA synthesis to maintain telomere length, inhibit telomere shortening, and prevent telomere self‐depletion [7, 8]. The enzymes include telomerase reverse transcriptase (TERT), telomerase RNA component (TERC), dyskerin protein, and other structural proteins. Mutations in telomerase‐related genes result in altered telomerase activity and telomere shortening. Telomere shortening and telomerase abnormalities are associated with the occurrence and development of ILD [9, 10]. There are still many challenges in the diagnosis and treatment of ILD, and the role of telomere shortening has been gradually emphasized.

## 2 Telomere Shortening Involving ILD

Because of numerous types of ILD and their similar clinical presentations, clinical diagnosis is difficult. For example, IPF and CTD‐ILD have similar clinical symptoms and pulmonary imaging features, making it difficult to make an accurate diagnosis in clinical practice. The diagnosis of diseases requires the cooperation of multiple clinicians, imaging specialists, and pathologists to confirm or revise the previous diagnosis with the information obtained. Therefore, effective methods for accurate diagnosis and differentiation are urgently needed in clinical practice to provide timely diagnosis and treatment for patients. It has been found that telomere shortening occurs in all patients with ILD, and telomere length and telomere shortening cells vary between different types of ILD [6]. The detection of telomere length may be helpful in the diagnosis and differentiation of ILD.

IPF is an ILD characterized pathologically by unusual interstitial pneumonia (UIP). Irreversible fibrosis is the hallmark of IPF, which is incurable and unclear in etiology. IPF is a diagnosis of exclusion, and telomere shortening has specificity in IPF patients, which has potential in the diagnosis and differentiation of IPF. In a Mendelian randomization study that included 1353 patients with IPF, Anna Duckworth found a correlation between telomere shortening in peripheral blood leukocytes and the development of IPF. For further comparison, Anna examined 11,413 patients with chronic obstructive pulmonary disease (COPD) and found that telomere shortening was not associated with the development of COPD [11]. One study performed quantitative polymerase chain reaction on peripheral blood leukocytes from IPF patients and found that the rate and extent of telomere shortening were higher in the IPF group than in normal controls [12, 13]. A study by Alder comparing telomere length in alveolar epithelial cells from healthy individuals, patients with disseminated IPF, and IPF patients with telomerase mutations also found telomere shortening in IPF patients [14]. Also, telomere lengths in alveolar epithelial cells and peripheral blood leukocytes from the same patients were compared, and a positive correlation was found between the presence of telomere shortening in peripheral leukocytes and in alveolar epithelial cells [14]. Snetselaar showed that telomere length was significantly shorter in IPF patients than in other ILD patients [6]. Telomere shortening has emerged as a clear risk factor for IPF and is expected to be valuable in the diagnosis and differentiation of IPF.

CTD‐ILD is a group of ILDs secondary to various types of connective tissue diseases, which are easily secondary in diseases such as rheumatoid arthritis (RA), systemic sclerosis (SSc), Sjogren's syndrome (SS), and systemic lupus erythematosus (SLE). There are numerous subtypes of CTD‐ILD, and telomere shortening has been found in patients with different types of CTD‐ILD. Antibodies for shelterin proteins in the serum of patients with CTD may be one of the reasons. TERT adds telomere repeat sequences to the end of telomeres. Shelterin proteins regulate the function of telomerase including TERF1 and TERF2. Brittany tested a group of CTD patients with RA, SSc, and myositis for Telomeric Repeat Binding Factor 1 (TERF1). It was found that the frequency of TERF1 positivity was higher in SSc patients than in RA, myositis, and healthy controls [15]. Telomere shortening in lymphocytes was present in SSc patients with TERF1 positivity, but the antibody was rarely detected in the sera of RA and myositis patients [15]. However, TERF1 is rarely detected in the sera of patients with RA and myositis.

ILD is the most common pulmonary manifestation of RA, accounting for 10%–20% of patients [16]. Approximately 10% of patients with RA die from ILD [17]. In Zeng's meta‐analysis, leukocyte telomere lengths were significantly shorter in RA patients than in controls [18]. Gamal reached similar conclusions, and he found that progressive telomere shortening occurred in the presence of increasing disease activity [19]. Natalini examined telomere length changes in peripheral blood cells of RA patients and found that although there was evidence of telomere shortening in RA‐ILD patients compared with RA patients without ILD, the causal relationship between telomere shortening and RA‐ILD was unknown [20].

SSc is less common than RA in patients with CTD, but pulmonary involvement is more common. ILD is the main manifestation of pulmonary involvement in SSc patients and is the leading cause of death by fa [21]. In a subset of SSc patients, TERF1 causes telomere shortening in peripheral lymphocytes and is associated with lung disease [16]. Patients with SSc had shorter age‐standardized telomere lengths compared with healthy controls, and SSc patients with ILD had shorter telomere lengths compared with those without ILD [22]. Liu examined the telomere length of peripheral blood cells in SSc‐ILD patients and found telomere shortening in peripheral leukocytes in SSc‐ILD patients compared with SSc patients without ILD [23]. In a study by Lakota, peripheral blood cells from SSc patients were divided into lymphocytes and granulocytes, which were examined separately for telomere length. The results showed that granulocytes from SSc‐ILD patients had normal telomere length and lymphocytes had shortened telomeres in contrast to IPF patients who had shortened telomeres in all peripheral blood cells [12].

The list of key studies on telomere shortening in cells from diverse ILDs, based on the updated literature, is presented in Table 1. Telomere shortening is present in both IPF patients and CTD‐ILD patients and is specific relative to healthy individuals. Telomere shortening is expected to be a key indicator for diagnosis and differential diagnosis among different types of ILD patterns.

**TABLE 1:** Telomere shortening in cells.

Disease	Cell	Key fundings
IPF	PBL [11]	Telomere shortening involving IPF
	Lymphocyte [12]	A contributory factor in IPF
	Granulocyte [12]	A contributory factor in IPF
	Leukocyte [13, 14]	Telomere shortening and telomerase mutations involving IPF
	Alveolar epithelial cell [14]	Telomere shortening in lung
SSc‐ILD	Lymphocyte [12, 15]	In lymphocytes while not in granulocytes
	Leukocyte [23]	Telomere shortening compared with SScs without ILD
RA‐ILD	Leukocyte [20]	Telomere shortening involving RA‐ILD
IIP	Leukocyte [14]	A risk factor for IIPs

Abbreviations: IIP, idiopathic interstitial pneumonia; IPF, idiopathic pulmonary fibrosis; PBL, peripheral blood lymphocyte; RA‐ILD, rheumatoid arthritis–associated interstitial lung disease; SS‐ILD, Sjogren's syndrome associated interstitial lung disease; SSc, systemic sclerosis; SSc‐ILD, systemic sclerosis associated interstitial lung disease.

## 3 Possible Mechanisms

The pathogenesis of ILD is complex and unclear, except for ILD with unique pathogenesis such as alveolar proteinosis and pulmonary Langerhans' cell histiocytosis. The pathogenesis of most ILD is a combination of pulmonary inflammation and pulmonary fibrosis.

Telomerase‐related mutations are associated with the development of ILD. It has been demonstrated that the presence of telomerase gene mutations in IPF patients promotes telomere shortening, which in turn causes replicative senescence of alveolar epithelial cells [24]. Cerri S et al. summarized several gene mutations and variations related to ILD. In addition to those related to telomere dysfunction, it included surfactant protein encoding genes, Mucin 5b gene that regulates mucosal defense, and Toll‐interacting protein gene [4]. Tsakiri found telomere shortening in peripheral blood leukocytes and alveolar epithelial cells of IPF patients. After genomic testing in IPF patients, he found that mutations in the TERC and TERT genes of telomerase could cause IPF [25]. The results of this study are consistent with the findings of Mushiroda [26]. Mutations in the telomerase TERT gene are the most common molecular defect in patients with IPF. TERT and TERC mutations are present in 8%–37% of patients with familial IPF, and mutations and loss of function in the TERT gene have been found in up to 15% of the families of familial IPF patients [13, 24]. Telomere shortening is present in approximately 25% of patients with non‐familial IPF, and TERT mutations are present in approximately 10% of patients with non‐familial IPF [13]. Telomere‐related mutations have also been detected in patients with CTD‐ILD [27, 28, 29]. In addition, genes that maintain telomere length include *NAF1*, *PARN*, *DKC1*, *TCAB1*, *RTEL1*, and *TINF2*, which are involved in telomere maturation and transport. Mutations in these genes cause telomere shortening and lead to pulmonary fibrosis. The incidence of genetic mutations in these genes in ILD patients is 0.3%, which is relatively rare [30, 31].

Most individuals carrying telomerase mutations have shortened telomeres, but there are other mechanisms that affect telomerase activity leading to telomere abnormalities and consequent pulmonary fibrosis [32]. The pathogenesis of IPF involves multiple theories. Based on histological features, abnormal alveolar epithelial damage‐repair may be the main etiology. When telomeres become extremely short, they activate the cellular DNA damage response leading to replicative senescence and pulmonary fibrosis in alveolar epithelial cells [33]. Different studies have confirmed that transforming growth factor‐beta (TGF‐β) inhibits telomerase gene activity via mothers against decapentaplegic homolog 3 (Smad3) in tumor cells and cultured mouse fibroblasts [34, 35, 36, 37, 38]. Smad3 is able to bind directly to the telomerase gene promoter and interfere with its transcription and translation [34]. Knockdown of the TGF‐β receptor in mouse lung epithelial cells inhibited the phosphorylation of Smad3, thereby reducing bleomycin‐induced lung fibrosis in mice [39]. The exact pathogenic mechanism between telomere abnormalities and pulmonary fibrosis is not fully understood. However, telomeres and telomerase play an important role in the development and progression of ILD.

## 4 Treatment

The treatments for ILD can be divided into two categories: pharmacological and non‐pharmacological treatments. Commonly used drugs include Pirfenidone and Nintedanib, but they can only delay the progressive destruction of alveoli and the accumulation of tissue fibrosis and cannot reverse the condition. Induction of cellular senescence in lung epithelial cells leads to significant pulmonary fibrosis in mice, suggesting that epithelial cell senescence is a key link in pulmonary fibrosis [40, 41]. Targeting telomerase and telomere function repair can inhibit the aging of alveolar epithelial cells and has broad therapeutic promise for the treatment of telomere abnormality–related pulmonary fibrosis. At present, relevant molecular therapies have demonstrated therapeutic value and gene therapy for telomeres has already been practiced.

Research has shown that estrogen receptors exist in the TERT gene promoter and estrogen can upregulate the expression of TERT and telomerase activity [42]. Danazol is an androgen derivative. There are case reports that two patients with telomeric abnormalities and TERC mutations were treated with danazol and alleviated anemia caused by telomeric abnormalities [43].

*GRN510* is a novel telomerase activator. le Saux used *GRN510* to treat pulmonary fibrosis in TERT gene mutant heterozygous mice whose pulmonary fibrosis was all induced by bleomycin. *GRN510* was found to increase telomerase activity 2–4 fold and inhibit the development of fibrosis, reducing inflammatory infiltration and collagen deposition. The condition was not improved when *GRN510* was combined with a telomerase inhibitor to treat mice, suggesting that the effect of *GRN510* is mediated by activation of telomerase [44].

The *PARN* gene is one of the genes that maintain telomere length by regulating the function of TERC. Another potential therapeutic pathway is the inhibition of PAP‐associated domain‐containing protein 5 (PAPD5) that opposes the *PARN* gene. Two potentially therapeutic PAPD5 inhibitors have been identified, *BCH001* and *RG7834*. *BCH001* lengthens telomeres in *PARN*‐mutated pluripotent stem cells from patients with short telomeres [45]. *RG7834* is a novel PAPD5 inhibitor that enhances telomerase activity in *PARN*‐knockout HeLa cells [46].

In recent years, gene therapy has gradually demonstrated therapeutic potential. Povedano used Tert gene therapy with adeno‐associated virus (AAV) as a vector to treat murine pulmonary fibrosis with telomere shortening, demonstrating that AAV‐*Tert* gene therapy can delay telomere shortening in lung epithelial cells and reduce pulmonary inflammation and decrease collagen deposition [41]. AAV‐*Tert* gene therapy also reduced lung fibroblasts in aged wild‐type mice and Ter‐knockout mice; slowed down telomere damage, senescence, and apoptosis in the lungs of both groups of mice; and significantly improved surface active substance damage in the lungs of both groups of mice [47]. Gene therapy may be a potential target for future IPF therapy, especially for patients with telomere shortening‐related pulmonary fibrosis. It has been proven to be a promising and promising direction for treatment.

## 5 The Assessment of ILD Prognosis

Identification of telomere shortening in ILD patients is important in predicting patient prognosis. Although IPF as an age‐increasing disease has an overall poorer prognosis in older patients, in the study by PLANAS, young IPF patients with telomere shortening had the worst prognosis, as telomere shortening was more severe in this group [48]. Similar results were obtained in the study by STUART; IPF patients with peripheral leukocytes having shorter telomere length had a poorer survival rate [49]. Patients with IPF who had acute exacerbation (AE) had shorter telomere lengths than patients with stable disease [50]. Therefore, the degree of telomere shortening would be a better prognostic factor than the patient's age. When the degree of telomere shortening is similar, the prognosis depends on the age of disease onset. The earlier the development of the pulmonary fibrosis process, the faster the pulmonary senescence and disease progress and the worse the prognosis will be [28]. Both the gender, age, and physiologic (GAP) staging and the composite physiologic index (CPI) are currently used clinically as tools to predict the risk of death in patients with IPF, but they are inadequate and have a greater margin of error for early stage patients. The discovery of new biomarkers such as the degree of telomere shortening may lead to better risk stratification.

Telomere length is just a predictor of survival in IPF patients, and it is not associated with survival in non‐IPF patients with interstitial lung disease. Stuart divided 370 ILD patients into IPF and non‐IPF groups. Telomere shortening was present in CTD‐ILD patients in the non‐IPF group, but the degree of telomere shortening was not significantly related to their survival [50]. The survival of CTD‐ILD patients was not related to prognosis and telomere length. It indicates that factors other than telomere length, such as the duration of environmental fibrosis exposure or the severity of the latent connective tissue disease, may be more relevant to disease progression and survival in CTD‐ILD patients.

## 6 Summary

The etiology and development of interstitial lung disease (ILD) involve a complex interplay of multiple contributing factors. From a genetic standpoint, telomere dysfunction, surfactant protein abnormalities, mutations in the MUC5B gene, TOLLIP gene variants, and other genetic and epigenetic alterations are closely linked to the pathogenesis of ILD [4]. Cerri S et al. provided an in‐depth review of the genetic and epigenetic factors influencing ILD, concluding that multiple genetic factors, including telomere‐related gene mutations, collectively contribute to the onset, progression, and clinical manifestations of the disease. Further research is warranted to explore the clinical translation and implementation of precision medicine for ILD.

**FIGURE 1:**
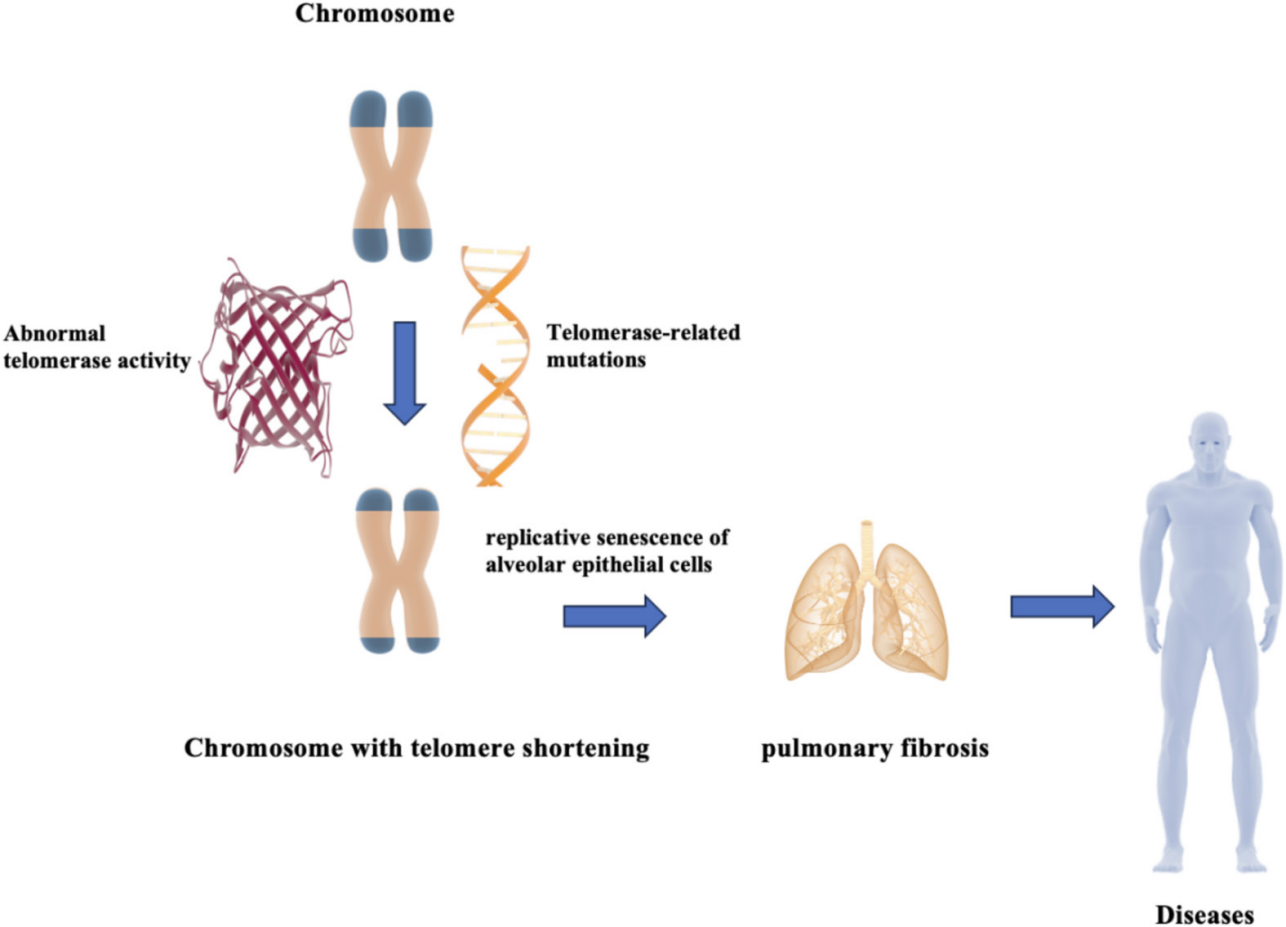
Schematic representation of the progression of pulmonary fibrosis due to the telomere shortening. Telomerase abnormalities such as related gene mutations lead to decrease in telomerase activity and telomere shortening. Individual cells undergoing continuous division exhibit telomere shortening. A successive shortening of the telomere results in dysfunctional telomeres. This induces replicative senescence of alveolar epithelial cells and ultimately progress to pulmonary fibrosis.

Telomere shortening and telomerase abnormalities play an important role in the progression of pulmonary fibrosis in ILD patients. Telomere shortening has become a clear risk factor in ILD patients and has been found in several ILD subtypes. Telomere abnormalities are involved in the development of ILD, and in addition to aging, altered telomerase activity and genetic mutations are also responsible. Telomerase gene mutations are commonly found in patients with IPF, and telomerase gene mutations have been found in family members of familial IPF patients. Figure 1 illustrates how telomere shortening can lead to pulmonary fibrosis. Detection of telomere length and telomerase gene will be helpful to assess the risk of asymptomatic patients. The degree of telomere shortening may be a useful indicator for evaluating the prognosis of ILD patients. The relationship between ILD and telomere shortening provides new ideas for treatment. Current treatments targeting telomerase and telomere function repair have shown therapeutic effects in animals. Further exploration of the relationship between telomeres, telomere‐related gene mutations, and ILD is needed, and further potential remains to be explored in the future in terms of progressive changes in the disease, therapeutic advice to patients, and prognostic risk.

## Author Contributions

Haonan Jin: investigation, formal analysis, writing – original draft. Jiamin Song: conceptualization, writing – original draft. Ronglin Gao: methodology, data curation, writing – original draft. Bingxian Sha: methodology, writing – original draft. Shengyuan Wang: investigation, writing – original draft. Peiming Luo: formal analysis, writing – original draft. Li Yu: conceptualization, project administration, funding acquisition, writing – review and editing. Xianghuai Xu: conceptualization, project administration, funding acquisition, writing – review and editing. Xuan Wang: conceptualization, supervision, project administration, funding acquisition, writing – review and editing.

## Ethics Statement

Not applicable.

## Consent

Not applicable.

## Conflicts of Interest

The authors declare no conflicts of interest.

## Data Availability

Data sharing not applicable to this article as no datasets were generated or analysed during the current study.

## References

[1] T. Gille and P. Laveneziana , “Cardiopulmonary Exercise Testing in Interstitial Lung Diseases and the Value of Ventilatory Efficiency,” European Respiratory Review 30, no. 162 (2021): 200355. 34853093 10.1183/16000617.0355-2020 PMC9489146

[2] H. R. Collard , C. J. Ryerson , T. J. Corte , et al., “Acute Exacerbation of Idiopathic Pulmonary Fibrosis. An International Working Group Report,” American Journal of Respiratory and Critical Care Medicine 194, no. 3 (2016): 265–275. 27299520 10.1164/rccm.201604-0801CI

[3] J. Gribbin , R. B. Hubbard , I. L. Jeune , et al., “Incidence and Mortality of Idiopathic Pulmonary Fibrosis and Sarcoidosis in the UK,” Thorax 61, no. 11 (2006): 980–985. 16844727 10.1136/thx.2006.062836 PMC2121155

[4] S. Cerri , E. Manzini , O. Nori , et al., “Genetic Risk Factors in Idiopathic and Non‐Idiopathic Interstitial Lung Disease: Similarities and Differences,” Medicina (Kaunas). 60, no. 12 (2024): 1967. 39768847 10.3390/medicina60121967 PMC11677115

[5] M. Wijsenbeek , A. Suzuki , and T. M. Maher , “Interstitial Lung Diseases,” Lancet 400, no. 10354 (2022): 769–786. 35964592 10.1016/S0140-6736(22)01052-2

[6] R. Snetselaar , C. H. M. Van Moorsel , K. M. Kazemier , et al., “Telomere Length in Interstitial Lung Diseases,” Chest 148, no. 4 (2015): 1011–1018. 25973743 10.1378/chest.14-3078

[7] C. Nicholls , A. R. Pinto , H. Li , et al., “Glyceraldehyde‐3‐Phosphate Dehydrogenase (GAPDH) Induces cancer Cell Senescence by Interacting With Telomerase RNA Component,” Proceedings of the National Academy of Sciences 109, no. 33 (2012): 13308–13313. 10.1073/pnas.1206672109 PMC3421169 22847419

[8] V. G. Sekaran , J. Soares , and M. B. Jarstfer , “Structures of Telomerase Subunits Provide Functional Insights,” Biochimica et Biophysica Acta (BBA)‐Proteins and Proteomics 1804, no. 5 (2010): 1190–1201. 19665593 10.1016/j.bbapap.2009.07.019

[9] E. J. Kim , H. R. Collard , and T. E. King, Jr. , “Rheumatoid Arthritis‐Associated Interstitial Lung Disease: The Relevance of Histopathologic and Radiographic Pattern,” Chest 136, no. 5 (2009): 1397–1405. 19892679 10.1378/chest.09-0444 PMC2818853

[10] N. Tanaka , J. S. Kim , J. D. Newell , et al., “Rheumatoid Arthritis‐Related Lung Diseases: CT Findings,” Radiology 232, no. 1 (2004): 81–91. 15166329 10.1148/radiol.2321030174

[11] A. Duckworth , M. A. Gibbons , R. J. Allen , et al., “Telomere Length and Risk of Idiopathic Pulmonary Fibrosis and Chronic Obstructive Pulmonary Disease: A Mendelian Randomisation Study,” Lancet Respiratory Medicine 9, no. 3 (2021): 285–294. 33197388 10.1016/S2213-2600(20)30364-7

[12] K. Lakota , V. S. Hanumanthu , R. Agrawal , et al., “Short Lymphocyte, But Not Granulocyte, Telomere Length in a Subset of Patients With Systemic Sclerosis,” Annals of the rheumatic diseases 78, no. 8 (2019): 1142–1144. 30679155 10.1136/annrheumdis-2018-214499

[13] J. T. Cronkhite , C. Xing , G. Raghu , et al., “Telomere Shortening in Familial and Sporadic Pulmonary Fibrosis,” American journal of respiratory and critical care medicine 178, no. 7 (2008): 729–737. 18635888 10.1164/rccm.200804-550OC PMC2556455

[14] J. K. Alder , J. J. Chen , L. Lancaster , et al., “Short Telomeres Are a Risk Factor for Idiopathic Pulmonary Fibrosis,” Proceedings of the National Academy of Sciences of the United States of America 105, no. 35 (2008): 13051–13056. 18753630 10.1073/pnas.0804280105 PMC2529100

[15] B. L. Adler , F. Boin , P. J. Wolters , et al., “Autoantibodies Targeting Telomere‐Associated Proteins in Systemic Sclerosis,” Annals of the rheumatic diseases 80, no. 7 (2021): 912–919. 33495152 10.1136/annrheumdis-2020-218918 PMC8217217

[16] Z. X. Yunt and J. J. Solomon , “Lung Disease in Rheumatoid Arthritis,” Rheumatic diseases clinics of North America 41, no. 2 (2015): 225–236. 25836639 10.1016/j.rdc.2014.12.004 PMC4415514

[17] A. Suzuki , Y. Ohosone , M. Obana , et al., “Cause of Death in 81 Autopsied Patients With Rheumatoid Arthritis,” Journal of rheumatology 21, no. 1 (1994): 33–36. 8151583

[18] Z. Zeng , W. Zhang , Y. Qian , et al., “Association of Telomere Length With Risk of Rheumatoid Arthritis: A meta‐Analysis and Mendelian Randomization,” Rheumatology (Oxford) 59, no. 5 (2020): 940–947. 31697380 10.1093/rheumatology/kez524

[19] R. M. Gamal , N. Hammam , M. M. Zakary , et al., “Telomere Dysfunction‐Related Serological Markers and Oxidative Stress Markers in Rheumatoid Arthritis Patients: Correlation With Diseases Activity,” Clinical Rheumatology 37 (2018): 3239–3246. 30328024 10.1007/s10067-018-4318-5

[20] J. G. Natalini , B. R. England , J. F. Baker , et al., “Associations Between Shortened Telomeres and Rheumatoid Arthritis‐Associated Interstitial Lung Disease Among U.S. Veterans,” Respiratory Medicine 201 (2022): 06943. 10.1016/j.rmed.2022.106943 PMC10120870 35947933

[21] Y. Allanore , R. Simms , O. Distler , et al., “Systemic Sclerosis,” Nature Reviews Disease Primers 1 (2015): 15002. 10.1038/nrdp.2015.2 27189141

[22] A. Usategui , C. Municio , E. G. Arias‐Salgado , et al., “Evidence of Telomere Attrition and a Potential Role for DNA Damage in Systemic Sclerosis,” Immunity & Ageing 19, no. 1 (2022): 7. 35086525 10.1186/s12979-022-00263-2 PMC8793167

[23] S. Liu , M. P. Chung , B. Ley , et al., “Peripheral Blood Leucocyte Telomere Length Is Associated With Progression of Interstitial Lung Disease in Systemic Sclerosis,” Thorax 76, no. 12 (2021): 1186–1192. 34272332 10.1136/thoraxjnl-2020-215918 PMC9262637

[24] M. Y. Armanios , J. J. Chen , J. D. Cogan , et al., “Telomerase Mutations in Families With Idiopathic Pulmonary Fibrosis,” New England Journal of Medicine 356, no. 13 (2007): 1317–1326. 17392301 10.1056/NEJMoa066157

[25] K. D. Tsakiri , J. T. Cronkhite , P. J. Kuan , et al., “Adult‐Onset Pulmonary Fibrosis Caused by Mutations in Telomerase,” Proceedings of the National Academy of Sciences 104, no. 18 (2007): 7552–7557. 10.1073/pnas.0701009104 PMC1855917 17460043

[26] T. Mushiroda , S. Wattanapokayakit , A. Takahashi , et al., “A Genome‐Wide Association Study Identifies an Association of a Common Variant in TERT With Susceptibility to Idiopathic Pulmonary Fibrosis,” Journal of medical genetics 45, no. 10 (2008): 654–656. 18835860 10.1136/jmg.2008.057356

[27] P. A. Juge , R. Borie , C. Kannengiesser , et al., “Shared Genetic Predisposition in Rheumatoid Arthritis‐Interstitial Lung Disease and Familial Pulmonary Fibrosis,” European Respiratory Journal 49, no. 5 (2017): 1602314. 28495692 10.1183/13993003.02314-2016

[28] C. A. Newton , K. Batra , J. Torrealba , et al., “Telomere‐Related Lung Fibrosis Is Diagnostically Heterogeneous but Uniformly Progressive,” European Respiratory Journal 48, no. 6 (2016): 1710–1720. 27540018 10.1183/13993003.00308-2016 PMC5433348

[29] E. Jönsson , L. Ljung , E. Norrman , et al., “Pulmonary Fibrosis in Relation to Genetic Loci in an Inception Cohort of Patients With Early Rheumatoid Arthritis From Northern Sweden,” Rheumatology (Oxford) 61, no. 3 (2022): 943–952. 33993221 10.1093/rheumatology/keab441 PMC8889303

[30] S. Petrovski , J. L. Todd , M. T. Durheim , et al., “An Exome Sequencing Study to Assess the Role of Rare Genetic Variation in Pulmonary Fibrosis,” American journal of respiratory and critical care medicine 196, no. 1 (2017): 82–93. 28099038 10.1164/rccm.201610-2088OC PMC5519963

[31] R. Borie , D. Bouvry , V. Cottin , et al., “Regulator of Telomere Length 1 (RTEL1) Mutations Are Associated With Heterogeneous Pulmonary and Extra‐Pulmonary Phenotypes,” European Respiratory Journal 53, no. 2 (2019): 1800508. 30523160 10.1183/13993003.00508-2018

[32] R. Faner , M. Rojas , W. Macnee , et al., “Abnormal Lung Aging in Chronic Obstructive Pulmonary Disease and Idiopathic Pulmonary Fibrosis,” American Journal of Respiratory and Critical Care Medicine 186, no. 4 (2012): 306–313. 22582162 10.1164/rccm.201202-0282PP

[33] D. C. Budd and A. M. Holmes , “Targeting TGFβ Superfamily Ligand Accessory Proteins as Novel Therapeutics for Chronic Lung Disorders,” Pharmacology & therapeutics 135, no. 3 (2012): 279–291. 22722064 10.1016/j.pharmthera.2012.06.001

[34] H. Li , D. Xu , J. Li , M. C. Berndt , and J. P. Liu , “Transforming Growth Factor beta Suppresses Human Telomerase Reverse Transcriptase (hTERT) by Smad3 Interactions With c‐Myc and the hTERT Gene,” Journal of biological chemistry 281, no. 35 (2006): 25588–25600. 16785237 10.1074/jbc.M602381200

[35] L. Cassar , H. Li , A. R. Pinto , C. Nicholls , S. Bayne , and J. P. Liu , “Bone Morphogenetic Protein‐7 Inhibits Telomerase Activity, Telomere Maintenance, and Cervical Tumor Growth,” Cancer research 68, no. 22 (2008): 9157–9166. 19010887 10.1158/0008-5472.CAN-08-1323

[36] L. Cassar , H. Li , F. X. Jiang , and J. P. Liu , “TGF‐beta Induces Telomerase‐Dependent Pancreatic Tumor Cell Cycle Arrest,” Molecular and cellular endocrinology 320, no. 1–2 (2010): 97–105. 20138964 10.1016/j.mce.2010.02.002

[37] A. Lacerte , J. Korah , M. Roy , X. J. Yang , S. Lemay , and J. J. Lebrun , “Transforming Growth Factor‐beta Inhibits Telomerase Through SMAD3 and E2F Transcription Factors,” Cell Signal 20, no. 1 (2008): 50–59. 17881189 10.1016/j.cellsig.2007.08.012

[38] B. Hu , D. C. Tack , T. Liu , Z. Wu , M. R. Ullenbruch , and S. H. Phan , “Role of Smad3 in the Regulation of Rat Telomerase Reverse Transcriptase by TGFbeta,” Oncogene 25, no. 7 (2006): 1030–1041. 16205635 10.1038/sj.onc.1209140

[39] M. Li , M. S. Krishnaveni , C. Li , et al., “Epithelium‐Specific Deletion of TGF‐β Receptor Type II Protects Mice From Bleomycin‐Induced Pulmonary Fibrosis,” Journal of clinical investigation 121, no. 1 (2011): 277–287. 21135509 10.1172/JCI42090 PMC3007138

[40] C. Yao , X. Guan , G. Carraro , et al., “Senescence of Alveolar Type 2 Cells Drives Progressive Pulmonary Fibrosis,” American journal of respiratory and critical care medicine 203, no. 6 (2021): 707–717. 32991815 10.1164/rccm.202004-1274OC PMC7958503

[41] J. M. Povedano , P. Martinez , R. Serrano , et al., “Therapeutic Effects of Telomerase in Mice With Pulmonary Fibrosis Induced by Damage to the Lungs and Short Telomeres,” Elife 7 (2018): e31299. 29378675 10.7554/eLife.31299 PMC5818250

[42] S. Bayne and J. P. Liu , “Hormones and Growth Factors Regulate Telomerase Activity in Ageing and Cancer,” Molecular and cellular endocrinology 240, no. 1–2 (2005): 11–22. 16005142 10.1016/j.mce.2005.05.009

[43] A. Islam , S. Rafiq , M. Kirwan , et al., “Haematological Recovery in Dyskeratosis Congenita Patients Treated With Danazol,” British journal of haematology 162, no. 6 (2013): 854–856. 23782100 10.1111/bjh.12432

[44] C. J. Le Saux , P. Davy , C. Brampton , et al., “A Novel Telomerase Activator Suppresses Lung Damage in a Murine Model of Idiopathic Pulmonary Fibrosis,” PLoS ONE 8, no. 3 (2013): e58423. 23516479 10.1371/journal.pone.0058423 PMC3597721

[45] N. Nagpal , J. Wang , J. Zeng , et al., “Small‐Molecule PAPD5 Inhibitors Restore Telomerase Activity in Patient Stem Cells,” Cell Stem Cell 26, no. 6 (2020): 896–909.e8. 32320679 10.1016/j.stem.2020.03.016 PMC7275922

[46] S. Shukla , H. C. Jeong , C. M. Sturgeon , R. Parker , and L. F. Z. Batista , “Chemical Inhibition of PAPD5/7 Rescues Telomerase Function and Hematopoiesis in Dyskeratosis Congenita,” Blood advances 4, no. 12 (2020): 2717–2722. 32559291 10.1182/bloodadvances.2020001848 PMC7322949

[47] S. Piñeiro‐Hermida , C. Autilio , P. Martínez , F. Bosch , J. Pérez‐Gil , and M. A. Blasco , “Telomerase Treatment Prevents Lung Profibrotic Pathologies Associated With Physiological Aging,” Journal of Cell Biology 219, no. 10 (2020): e202002120. 32777016 10.1083/jcb.202002120 PMC7659728

[48] L. Planas‐Cerezales , E. G. Arias‐Salgado , I. Roldán , et al., “Predictive Factors and Prognostic Effect of Telomere Shortening in Pulmonary Fibrosis,” Respirology 24, no. 2 (2019): 146–153. 30320420 10.1111/resp.13423

[49] B. D. Stuart , J. S. Lee , J. Kozlitina , et al., “Effect of Telomere Length on Survival in Patients with Idiopathic Pulm Onary Fibrosis: An Observational Cohort Study with Independent Validation,” Lancet Respiratory Medicine 2, no. 7 (2014): 557–565. 24948432 10.1016/S2213-2600(14)70124-9 PMC4136521

[50] I. Tomos , A. Karakatsani , E. D. Manali , et al., “Telomere Length Across Different UIP Fibrotic‐Interstitial Lung Diseases: A Prospective Greek Case‐Control Study,” Pulmonology 28, no. 4 (2022): 254–261. 33358512 10.1016/j.pulmoe.2020.11.005

